# Boldness predicts foraging behaviour, habitat use and chick growth in a central place marine predator

**DOI:** 10.1007/s00442-024-05557-4

**Published:** 2024-05-13

**Authors:** Jorge M. Pereira, Jaime A. Ramos, Filipe R. Ceia, Lucas Krüger, Ana M. Marques, Vitor H. Paiva

**Affiliations:** 1 Department of Life Sciences, University of Coimbra, MARE - Marine and Environmental Sciences Centre / ARNET - Aquatic Research Network, Calçada Martim de Freitas, 3000-456 Coimbra, Portugal; 2 Instituto Antártico Chileno, Plaza Muñoz Gamero 1055, 620 000 Punta Arenas, Chile; 3 Instituto Milenio Biodiversidad de Ecosistemas Antárticos y Subantárticos (BASE), Las Palmeras 3425, Ñuñoa, Santiago, Chile

**Keywords:** Cory’s shearwater, Foraging flexibility, Personality, Resource acquisition, Resource predictability

## Abstract

Animal personality can shape individual’s fitness. Yet, the mechanistic relationship by which individual’s personality traits lead to variations in fitness remains largely underexplored. Here, we used novel object tests to measure boldness of chick-provisioning Cory’s shearwaters (*Calonectris borealis*) from a coastal colony off west Portugal, and deployed GPS loggers to study their at-sea behaviour and distribution. We then tested whether boldness predicts individual differences in adult’s trophic ecology and variations in chick growth, to assess potential implications of personality-specific foraging behaviours. Foraging effort was higher for shyer than for bolder individuals, which, during short forays, exhibited larger foraging ranges, and foraged in regions of higher and more variable bathymetry. This suggests that nearby the colony bolder individuals expanded their foraging area to maximize resource acquisition and increase the probability of foraging success. When endeavouring to longer distances, bolder individuals exhibited comparably shorter foraging ranges and targeted low bathymetry regions, likely with enhanced prey availability, while shyer individuals exhibited much larger foraging ranges indicating greater flexibility when foraging in oceanic realms. Despite such differences between bolder and shyer individuals their isotopic niches were similar. Yet, chicks raised by bolder parents grew at a faster rate than those raised by shyer parents. Together, our results suggest that differences in resource acquisition strategies could play a key role through which individual’s boldness may influence breeding performance, even when individuals have similar isotopic preferences.

**Supplementary Information:**

The online version contains supplementary material available at 10.1007/s00442-024-05557-4.

## Introduction

There is increasing evidence that differences in both foraging and feeding strategies within a population are widespread (Bolnick et al. 2003). Such within-population differences can vary with extrinsic factors such as intra-specific competition (Sheppard et al. 2021) and predictability of environmental conditions (Woo et al. 2008). Considering that global change is rapidly deteriorating environmental conditions and altering food webs (Hoegh-Guldberg and Bruno 2010), understanding the mechanisms underlying within-population differences in both foraging and feeding strategies is crucial to predict how species will cope with future environmental changes.

The majority of studies assessing within-population differences in wild animals focused on diet (Bolnick et al. 2003; Araújo et al. 2011; Ceia and Ramos 2015; Phillips et al. 2017). In turn, diet differences between populations will likely be linked to other ecological traits over short-term periods, such as in foraging behaviour or habitat selection by the individuals of each population (Carneiro et al. 2017; Shaw 2020). Optimal foraging theory broadly predicts that central-place foragers should adjust their foraging behaviour to maximize foraging efficiency and consequently increase fitness (MacArthur and Pianka 1966). Foraging efficiency has been suggested to play a key role through which spatial and habitat specialization relate to reproductive success (Lescroël et al. 2010). For instance, foraging efficiency increased with foraging site fidelity in Magellanic penguins (*Spheniscus magellanicus*) (Rebstock et al. 2022) and habitat specialization of Herring gulls (*Larus argentatus*) (van den Bosch et al. 2019), improving the breeding performance in both species.

Individual foraging specialization is expected to be widespread in marine environments (Switzer 1993) and may arise as a consequence of exploiting predictable oceanographic conditions, such as bathymetric features (e.g., shelf edges and seamounts), oceanic fronts and coastal upwelling, often leading to spatio-temporally predictable prey patches (Irons 1998; Weimerskirch 2007; Riotte-Lambert and Matthiopoulos 2020). For example, foraging site fidelity in chick-rearing Northern gannets (*Morus bassanus*) was previously reported to be higher in waters with predictable resource availability in the North Sea, particularly in shelf break areas, than in the highly dynamic waters of the Celtic Sea (Hamer et al. 2001). In predictable environments, foragers should benefit from previous knowledge of food availability gained by the repeatable use of high-productive prey patches (Piper 2011; Wakefield et al. 2015). Under these conditions, individuals are expected to develop specialized foraging behaviours enabling them to locate prey more efficiently and consequently increase their fitness and reproductive success (Switzer 1993; Rebstock et al. 2022).

Recent studies show that personality traits, foraging behaviour and individual foraging specialization can covary in the wild (Toscano et al. 2016; Spiegel et al. 2017). For instance, personality traits have been linked to individual foraging movement (Patrick and Weimerskirch 2014), resource acquisition strategies (Patrick et al. 2017; Traisnel and Pichegru 2019), and foraging site fidelity (Harris et al. 2020a) in breeding seabirds, with consequences for individual fitness (Patrick and Weimerskirch 2014, 2015; Harris et al. 2020b). However, the mechanistic links by which individual differences in personality may lead to variations in reproductive success remains largely underexplored (Smith and Blumstein 2008). Thus, differences in foraging behaviour and the degree of individual foraging specialization may represent important, yet poorly studied, pathways through which personality may influence reproductive success.

In this study, we relate seabird individual boldness with foraging behaviour, spatial aspects of foraging, diet and chick growth rate using Cory’s shearwaters (*Calonectris borealis*) foraging in the west coast of Portugal. Prey-resources available to Cory’s shearwaters breeding at Berlenga Island are influenced by the major summer upwelling occurring along the western coast of the Iberian Peninsula (Paiva et al. 2010c). Most Cory’s shearwaters from this coastal population engage in short foraging trips, searching for epipelagic fish in predictable prey patches near the colony, especially during the chick-rearing period (Paiva et al. 2010a, b; Pereira et al. 2022). Thus, long foraging trips to offshore pelagic waters are comparatively less frequent, though they become more frequent under scenarios of low food availability (Paiva et al. 2013, 2017; Pereira et al. 2020). We used a combination of GPS tracking, oceanographic characteristics, stable isotopes and chick growth data of bold and shy chick-provisioning Cory’s shearwaters to address the following three hypothesis:

1. Bolder birds should forage more efficiently, with shorter time spent foraging per trip (i.e., lower foraging effort) as they generally favour a more risk-taking behaviour (Sih et al. 2004; Wolf et al. 2007; Dammhahn and Almeling 2012). In contrast, shyer individuals, which are typically more risk-averse (Sloan Wilson et al. 1994), should search for food more using mostly an explorative behaviour (i.e., higher foraging effort) to minimize the risk of unsuccessful foraging (Patrick et al. 2017; Jeffries et al. 2021).
2. Bolder individuals should present stronger specialization in foraging habitat, which would show in more consistent selection of either short trips to coastal habitats or long trips to pelagic habitats, whereas shyer individuals should be more variable in foraging habitat use (Cockrem 2022).
3. Bolder birds are expected to be more successful in environments where resources are spatio-temporal predictable (Dingemanse and Réale 2005; Cockrem 2007). Accordingly, bolder individuals should ingest higher trophic level prey in near-shore prey patches (i.e., closer to the colony) and thus exhibit comparably higher nitrogen and carbon isotopic values, respectively. This should enable them to feed and raise chicks more successfully (at a faster growth rate) when compared to shyer individuals.

## Material and methods

### Study system

We studied Cory’s shearwaters during two consecutive breeding seasons (2017–2018) at Berlenga Island on the west coast of Portugal (39°23′ N, 9°36′ W), during the mid chick-rearing (August–September). All individuals (*N* = 35) were breeding adults of unknown age. Only one individual was studied across the two years. Each one of these 35 individuals was equipped with Global Positioning System (GPS) loggers, sexed according to vocalizations (i.e., higher pitched vocalizations of males when compared to females; Bretagnolle and Lequette 1990), tested for boldness, blood sampled for stable isotopes analysis, and assessed its breeding performance using chick growth rate (see Table 1 for details on sample sizes).

**Table 1:** Study period, sample sizes and tracking details for Cory’s shearwaters (*Calonectris borealis*) from Berlenga Island, during the chick-rearing period (2017–2018). Tracked birds were tested for boldness and blood sampled for stable isotope analysis

	2017	2018	Total
*Experimental design*			
Study period	10 Aug–17 Sep	17 Aug–24 Sep	
*N* tracked birds	10	25	35
*N* females	4	15	19
*N* males	6	10	16
*N* monitored chicks	10	25	35
*Tracking details*			
Tracking duration (days)	21.4 ± 4.2	9.4 ± 2.9	12.8 ± 6.4
Trip duration (days)	2.1 ± 0.4	1.9 ± 0.5	2.0 ± 0.5
Maximum distance from colony (km)	151.8 ± 53.1	231.8 ± 307.0	208.8 ± 262.0
*N* of foraging trips	145	169	314
*N* of short trips	129	152	281
*N* of long trips	16	17	33

### Boldness

We measured adult Cory’s shearwaters’ boldness as the degree of response to a novel object, while at the nest. We followed a protocol previously used in our study population and described by Krüger et al. (2019) and Pereira et al. (2021). Individual’s response towards a novel object is correlated with boldness, and together form part of a risk-taking behaviour (Wolf et al. 2007). Thus, fewer movements or non-aggressive behaviours towards the object at the nest are frequently interpreted as ‘shyer’ responses, whereas agitated behaviours and more interactions with the object are often interpreted as ‘bolder’ responses (Sih et al. 2004).

Briefly, we measured birds’ response to a LED headlamp (6.2 × 4.0 × 3.5 cm; Lighting EVER®) coupled to a Campark Action HD waterproof camera (6.0 × 2.5 × 4.0 cm; Campark®) which was placed in the nest entrance for approximately 2 min. Following Krüger et al. (2019), we subsequently recorded the number of times an individual exhibited 6 mutually exclusive behaviours, spanning from less movements or response towards the object to behaviours associated with more body mobility or actions towards the camera, bird: (1) moves the head without moving from its position; (2) gives a short and sudden jerking or convulsive-like movement (spasm); (3) moves away from the object; (4) bird opens and closes the bill without charging in the direction of the object (snap); (5) makes contact with the object (peck); and/or (6) stands up off chicks. Repeated tests from the same individual were always conducted on different days. All the 35 individuals used in this study were tested for boldness: 5 individuals were tested once, 9 were tested twice, 17 were tested three times and 4 were tested more than three times (totalling 91 videos over the two years).

To obtain a single estimate of boldness per individual, we applied a non-metric multidimensional scaling (NMDS) analysis to assign the 6 recorded behaviours along a boldness-shyness continuum (first NMDS axis—NMDS 1 values; see Supplementary Material Table S1 for variable loadings) using the ‘*vegan*’ R package (Oksanen et al. 2013). For visualization purposes only, birds were categorized as either ‘shyer’ (small values) or ‘bolder’ (large values) based on the median boldness scores, resulting in 17 shy individuals and 18 bold individuals. Boldness was previously shown to be repeatable within individuals and not influenced by sex in our study population of Cory’s shearwaters (Krüger et al. 2019). To confirm this repeatability, we estimated the adjusted repeatability (Nakagawa and Schielzeth 2010) of NMDS1 using the ‘*rptR*’ R package (Stoffel et al. 2017), including fixed effects to adjust for test date and test number. Lastly, we further tested for sex differences in boldness estimates using a generalized linear model (GLM) with sex as fixed effect.

### GPS tracking

GPS loggers (CatLog2; Perthold Engineering) were attached to the birds’ four central tail feathers using TESA® tape and retrieved two weeks later after several consecutive trips (see results) (for further details on tag deployment, please see Supplementary Material 1). Tracking data were first filtered to remove positions within a 1 km radius of the colony to reduce the influence of rafting behaviour close to the colony. Next, we identified individual foraging trips and calculated the trip duration (total time spent on a foraging trip, in days) and the maximum distance from colony (distance between the furthermost location of the trip and the breeding colony, in km). Tracking datasets were partitioned in short (≤2 days, ≤100 km) and long trips (>2 days, >100 km) based on histograms of the frequency of occurrence of trip duration and maximum distance from colony reached on each foraging trip (Supplementary Material Figure S1).

To characterize birds’ foraging behaviour during each excursion, we used the ‘*Expectation–Maximisation binary Clustering*’ algorithm from package ‘*EmbC*’ (Garriga et al. 2016) to classify each GPS position in either (1) travelling (high velocity, low tortuosity), (2) extensive search (high velocity, high tortuosity), (3) intensive search (low velocity, high tortuosity) or (4) resting (low velocity, low tortuosity). Intensive search (i.e., decreases in velocity and abrupt changes in bird trajectory) are related with area-restricted search (ARS) behaviour, whereas extensive search reflects important turns with a steady speed, which can be interpreted as a displacement between areas of intensive search behaviour. Intensive and extensive search behaviours were then grouped as “foraging” positions, and considered to represent small- and large-scale ARS, respectively (Weimerskirch et al. 2007; Clay et al. 2019). We further calculated the percentage of time individuals spent in each behavioural state during each foraging trip.

Kernel density estimates were generated for the foraging positions only (i.e., intensive and extensive search) using the ‘*adehabitatHR*’ R package (Calenge 2006). We further calculated the area (in km^2^) of 50% kernel Utilization Distributions (UDs) between trips of each individual per year (a proxy for foraging spatial consistency; Cerveira et al. 2020). We also used the ‘*kerneloverlap*’ function to calculate the Home Range (HR) overlap index, where 0 indicates no spatial overlap and 1 indicates complete spatial overlap in the 50% kernel UDs at individual’s trip level. Kernel density estimates were generated using a grid size of 0.08° (approximately 8 km) to match the coarsest grid of the environmental variables. The most appropriate smoothing parameter (*h*) was calculated using the R package ‘*track2KBA*’ (Beal et al. 2021), as the average value of area-restricted search (ARS) behaviour exhibited across short and long foraging trips for each year (approximately 10 km).

### Environmental variables

We examined habitat use of Cory’s shearwaters in relation to one static (bathymetry), and three dynamic environmental variables (chlorophyll-*a* concentration; ocean mixed layer depth; and eddy kinetic energy). These environmental variables were chosen following previous studies demonstrating their influence on the foraging distribution of individuals from our study population or in other closely related species (see Supplementary Material Table S2 for details on data sources, data resolution and rationale for the inclusion of each variable). Environmental variables were extracted for the foraging positions only (i.e., GPS positions where individuals engaged in intensive and extensive search behaviours). Prior to any statistical analysis, we inspected collinearity among environmental variables using variance inflation factors (VIF values; see Supplementary Material Table S3) and the R package ‘*usdm*’ (Naimi et al. 2014). Initially, we downloaded sea surface temperature (°C) and sea surface height (m), but we decided to exclude these two variables from further analysis because they were highly collinear (VIF values ≥2.5; Johnston et al. 2018) with other predictors (see Supplementary Material Table S3), and when running univariate models with each of them, their Akaike’s information criterion corrected for sample sizes (AICc) values were comparably higher than their colinear counterparts.

### Stable isotopes analyses

Blood samples (approximately 0.5–1.0 ml) were collected from the metatarsal or brachial vein of individual birds after logger retrieval. Within 2–4 h from sampling, blood samples were separated into plasma and red blood cell fractions using a centrifuge and frozen at –20 °C until preparation for stable isotope analysis. Prior to stable isotope analysis, samples were defrosted, homogenized and dried overnight at 40 °C. Lipids removal was performed with successive rinses in a 2:1 chloroform/methanol solution, to avoid the high lipid concentrations of plasma that can result in depleted ^13^C values (Cherel et al. 2005). We then analyzed stable isotope ratio values for nitrogen (*δ*^15^N) and carbon (*δ*^13^C) in the red blood cells and plasma to study the trophic ecology of each bird during each year. Red blood cells have a turnover rate of a few weeks and plasma of a few days (Hobson 2005), reflecting the assimilated diet over the previous 4–6 weeks and the last trips before sampling (and logger retrieval), respectively. Laboratory procedures for stable isotope analysis are described in Supplementary Material 2.

### Chick growth

Chicks from each of the 35 adults used in this study were weighed (to the nearest 5 g) every two days during the linear growth period using a Pesola® spring balance. We calculated the linear growth rate (g day^−1^) of each chick from the slope of the regression line of chick body mass during the linear growth period, between 10 and 40 days of age (Ramos et al. 2003).

### Statistical analysis

All the statistical analysis was based on a sample size of 35 individuals, as all these birds were tracked with GPS loggers, tested for boldness, sampled for stable isotopes analysis, and assessed its breeding performance using chick growth rate (Table 1).

At trip level, we used generalized linear mixed models (GLMMs) to test whether boldness predicted: (1) individual’s at-sea foraging behaviour (time spent foraging and resting); and (2) habitat use (area of 50% kernel UDs, overlap in 50% kernel UDs, bathymetry, chlorophyll-*a* concentration, ocean mixed layer depth and eddy kinetic energy), during short and long trips. Separate models were fitted with trip level average for each of the previous variables as a response variable in each model, resulting in 8 models (Table 2). In all GLMMs we began by including boldness, trip type (short vs. long trips), sex, (females vs. males), year (2017 vs. 2018), and the two-way interactions between boldness and trip type, boldness and sex, and boldness and year as fixed effects. Trip identity nested within individual identity was fitted as a random effect to control for pseudo-replication of multiple trips per individual.

**Table 2:** Description of the best-supported generalized linear models and generalized linear mixed models (GLMs and GLMMs with the lowest Akaike’s information criterion corrected for sample sizes—AICc values) explaining foraging behaviour, habitat use, trophic ecology and chick growth of Cory’s shearwaters as a function of individual’s boldness (NMDS 1 values), trip type (short vs. long trips), sex, (females vs. males) and year (2017 vs. 2018)

Research question	Model type	Response variables	Explanatory variables	Random effects	AICc
Influence of boldness on foraging behaviour	GLMM—beta distribution	Time spent foraging (%)	Boldness × Trip type + Sex + Year	Bird ID/Trip ID	757.9
	GLMM—beta distribution	Time spent resting (%)	Boldness + Trip type + Year	Bird ID/Trip ID	512.6
Influence of boldness on habitat use	GLMM—tweedie distribution	Area of 50% kernel UDs (km^2^)	Boldness × Trip type	Bird ID/Trip ID	4552.4
	GLMM—beta distribution	Overlap in 50% kernel UDs (%)	Trip type + Year	Bird ID/Trip ID	883.2
	GLMM—tweedie distribution	Bathymetry (m)	Boldness × Trip type + Sex	Bird ID/Trip ID	4017.6
	GLMM—tweedie distribution	Chlorophyll-*a* concentration (mg m^−3^)	Trip type + Year	Bird ID/Trip ID	851.0
	GLMM—tweedie distribution	Ocean mixed layer thickness (m)	Trip type + Sex + Year	Bird ID/Trip ID	769.2
	GLMM—tweedie distribution	Eddy kinetic energy (cm^−2^ s^−2^)	Trip type + Year	Bird ID/Trip ID	112.1
Influence of boldness on trophic ecology	GLM—gaussian distribution	*δ*^13^C in red blood cells (‰)	Sex + Year	–	25.9
	GLM—gaussian distribution	*δ*^15^N in red blood cells (‰)	Sex + Year	–	30.3
	GLM—gaussian distribution	*δ*^13^C in plasma (‰)	Sex + Year	–	53.0
	GLM—gaussian distribution	*δ*^15^N in plasma (‰)	Sex + Year	–	39.9
Influence of boldness on chick growth	GLM—gaussian distribution	Linear growth rate (g day^−1^)	Boldness	–	190.3

At individual level, we used GLMs to test whether boldness was associated to: (1) trophic ecology of adults; and (2) chick growth. Also here, separate models were fitted with the carbon (*δ*^13^C) and nitrogen stable isotope ratios (*δ*^15^N) in the red blood cells and plasma, and the chick linear growth rate as response variable in each model, resulting in 5 models (Table 2). In all GLMs, we also began by including boldness, sex, year, and the two-way interactions between boldness and sex, and boldness and year as fixed effects.

In all GLMMs and GLMs, we started with the full model interactions. The least significant fixed-effect terms were then removed sequentially via backward stepwise selection to obtain the models with the lowest value of AICc (Table 2). We then checked the models that performed best for normality and homogeneity by visual inspection of residual plots using the ‘*performance*’ R package (Lüdecke et al. 2021). For each model, we used the most appropriate statistical distributions that better fitted the data to approximate normality (Table 2). We extracted and plotted predicted values and confidence intervals (CI) from the best-supported models (models with the lowest AICc values) using the “*ggpredict*” function within the “*ggeffects*” R package (Lüdecke 2018). GLMMs were computed using the “*glmmTMB*” R package (Brooks et al. 2017). All statistical analysis were carried out in R v. 4.0.5 (R Core Team 2022). All data are presented as mean ± SD (standard deviation) unless otherwise stated. Differences were considered statistically significant at *p* ≤ 0.05.

## Results

### Boldness

Boldness scores (NMDS 1 values) ranged from −0.49 to 0.61, with small values representing instances when birds exhibited little reactions or non-aggressive behaviours towards the object (interpreted as ‘shyer’ responses), and large values representing instances when birds attacked the object or raised up to protect the chicks (interpreted as ‘bolder’ responses; see Supplementary Material Table S1 for variable loadings). Cory’s shearwaters were repeatable in their response to the novel object over the two years of study (*R* = 0.30, CI: 0.22–0.67, *p* < 0.001). We found no sex-differences in boldness scores (*F*_1,89_ = 1.62, *p* = 0.21).

### Foraging trip characteristics

We recorded a total of 314 foraging trips made by 35 chick-rearing Cory’s shearwater adults (Supplementary Material Figure S2), averaging 13.5 ± 2.8 trips per bird during 2017 (range: 8–18 trips) and 6.8 ± 2.3 trips per bird during 2018 (range: 3–12 trips). Over the two breeding seasons, Cory’s shearwaters made trips ranging up to 9 days in duration, and up to 1318 km from the colony. From the total trips, Cory’s shearwaters engaged mostly in short foraging trips (89.5%) and to a lesser extent in long foraging trips (10.5%; Table 1). Although tracking duration differed significantly between years (*F*_1,312_ = 72.86, *p* < 0.001), foraging trip characteristics, such as trip duration (*F*_1,33_ = 0.60, *p* = 0.45), maximum distance from colony (*F*_1,33_ = 0.67, *p* = 0.42) and proportion of long trips (*F*_1,33_ = 0.08, *p* = 0.79) were similar between years (Table 1).

### Effect of boldness on at-sea foraging behaviour

Boldness was associated to a decrease of 7% in the time spent foraging, ranging from 49% (95% CI = 45–54%) for shyer individuals to 42% (95% CI = 39–45%) for bolder individuals (Table 3). Although not significant (Table 3), time spent foraging tended to decrease with boldness during short trips, but not during long trips (Supplementary Material Figure S3). In contrast, boldness was associated to an increase of 7% in the time spent resting, ranging from 25% (95% CI = 21–30%) for shyer individuals to 32% (95% CI = 29–35%) for bolder individuals (Table 3). We found no evidence for interacting effects of boldness with sex, or boldness with year in the time spent in any behavioural state (Table 2).

**Table 3:** Results of the best-supported generalized linear mixed models (GLMMs with the lowest Akaike’s information criterion corrected for sample sizes—AICc values) explaining foraging behaviour and habitat use of Cory’s shearwaters as a function of individual’s boldness (NMDS 1 values), trip type (short vs. long trips), sex, (females vs. males) and year (2017 vs. 2018)

Models and explanatory variables	Estimate ± SE	GLMM (*χ*^2^_1,312_)	*p*
*Time spent foraging (%)*			
Boldness	−0.27 ± 0.09	7.02	**0.001**
Trip type (long trips)	−0.23 ± 0.06	10.71	**<0.001**
Boldness x Trip type (long trips)	0.31 ± 0.16	3.62	0.06
Sex (males)	0.11 ± 0.05	4.62	**0.03**
Year (2018)	−0.22 ± 0.06	11.58	**<0.001**
*Time spent resting (%)*			
Boldness	0.30 ± 0.13	5.29	**0.02**
Trip type (long trips)	−0.30 ± 0.10	10.11	**0.001**
Year (2018)	0.39 ± 0.09	16.61	**<0.001**
*Area of 50% kernel UDs (km*^*2*^*)*			
Boldness	0.21 ± 0.10	1.29	**0.03**
Trip type (long trips)	1.00 ± 0.08	144.07	**<0.001**
Boldness x Trip type (long trips)	−1.00 ± 0.21	22.97	**<0.001**
*Overlap in 50% kernel UDs (%)*			
Trip type (long trips)	−0.83 ± 0.12	49.27	**<0.001**
Year (2018)	−0.18 ± 0.07	6.53	**0.01**
*Bathymetry (m)*			
Boldness	0.71 ± 0.28	4.90	**0.01**
Trip type (long trips)	1.57 ± 0.20	58.61	**<0.001**
Boldness x Trip type (long trips)	−1.36 ± 0.55	6.21	**0.01**
Sex (males)	−0.56 ± 0.24	5.49	**0.02**
*Chlorophyll-a concentration (mg m*^*−3*^*)*			
Trip type (long trips)	−0.26 ± 0.12	4.64	**0.03**
Year (2018)	−0.75 ± 0.16	20.74	**<0.001**
*Ocean mixed layer thickness (m)*			
Trip type (long trips)	0.03 ± 0.01	6.05	**0.01**
Sex (males)	−0.03 ± 0.01	5.12	**0.02**
Year (2018)	−0.06 ± 0.01	26.34	**<0.001**
*Eddy kinetic energy (cm*^*−2*^* s*^*−2*^*)*			
Trip type (long trips)	−0.31 ± 0.11	7.83	**0.001**
Year (2018)	2.62 ± 0.13	395.13	**<0.001**

Coefficients of categorical fixed effects (i.e., all except "Boldness") were calculated relative to their reference: long trips (Trip type), males (Sex), and 2018 (Year). Each model included trip identity nested within the individual as a random effect

Differences were statistically significant when *p* ≤ 0.05 (in bold)

### Effect of boldness on habitat use

**Fig. 1:**
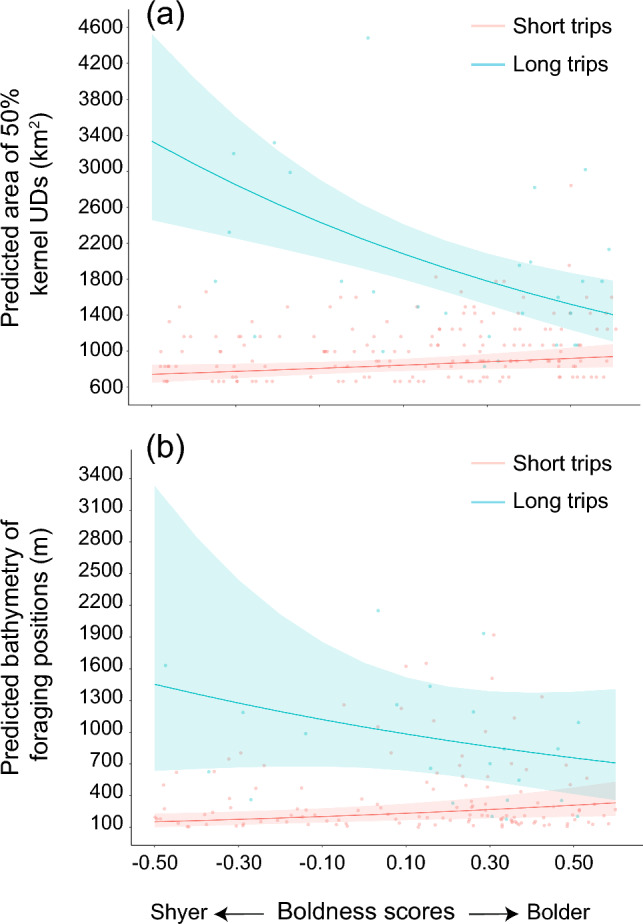
Mean predicted effects of individual’s boldness (NMDS 1 values) on the **a** area of 50% kernel Utilization Distributions (UDs; km^2^) interacting with trip type (short vs. long trips); and **b** bathymetry of foraging positions (m) interacting with trip type. *Red lines* represent short trips and *blue lines* represent long trips. Lower NMDS 1 values represent ‘shyer’ responses and higher NMDS 1 values represent ‘bolder’ responses toward the object. Predicted values (*points*), regression lines and 95% confidence intervals (*shaded areas*) were extracted from the best-supported models (models with the lowest Akaike’s information criterion corrected for sample sizes—AICc values; see Table 2). See Table 3 for model estimates, test statistics and *p* values (color figure online)

**Fig. 2.**
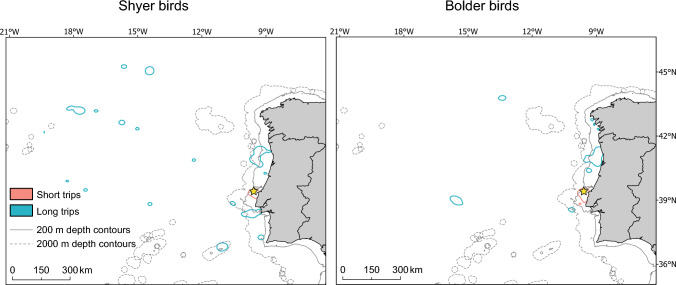
50% kernel Utilization Distributions (UDs) of shyer and bolder Cory’s shearwaters during short and long trips, in 2017–2018. For visualization purposes only, birds were categorized as either ‘shyer’ (*small values*) or ‘bolder’ (*large values*) based on the median boldness scores, resulting in 17 shy individuals and 18 bold individuals. The location of the breeding colony (Berlenga Island) is marked with a yellow star. Solid and dashed lines represent the 200 and 2000 m depth contours, respectively

Boldness was associated to an increase of 1.3 times in the size of foraging areas (Table 3), predicting larger foraging ranges for bolder individuals (predicted area of 50% kernel UDs: 940 km^2^, 95% CI = 820–1078 km^2^) than for shyer individuals (predicted area of 50% kernel UDs: 742 km^2^, 95% CI = 650–848 km^2^). However, when interacting with trip type, we found that boldness was associated to an increase in the size of foraging areas during short trips, but not during long trips (Fig. 1a). During long trips, boldness was associated to a decrease of 2.4 times in the area of 50% kernel UDs, respectively (Table 3; Fig. 1a), predicting smaller foraging areas for bolder individuals (predicted area of 50% kernel UDs: 1406 km^2^, 95% CI = 1108–1786 km^2^; Fig. 2) than for shyer individuals (predicted area of 50% kernel UDs: 3337 km^2^, 95% CI = 2459–4528 km^2^; Fig. 2). Interestingly, shyer individuals differed markedly in the size of foraging areas between short and long trips, whereas these differences were comparatively small for bolder individuals (Fig. 1a).

Boldness was associated to an increase of 2.2 times in the bathymetry of foraging positions (Table 3), predicting that bolder individuals foraged more often in regions of higher and more variable bathymetry (predicted bathymetry: 332 m, 95% CI = 207–532 m) than shyer individuals (predicted bathymetry: 153 m, 95% CI = 101–230 m). However, when interacting with trip type, we found that boldness was associated with deeper waters during foraging in short trips, but not in long trips (Fig. 1b). During long trips, boldness was associated to a two-fold decrease in the bathymetry (Table 3; Fig. 1b): bolder birds foraged more often in regions of lower bathymetry (predicted bathymetry: 710 m, 95% CI = 358–1408 m; Fig. 2), whereas shyer individuals foraged in regions of higher and more variable bathymetry (predicted bathymetry: 1454 m, 95% CI = 634–3334 m; Fig. 2). Moreover, shyer birds differed markedly in the bathymetry of foraging positions between short and long trips, whereas these differences were comparatively small for bolder individuals (Fig. 1b).

We did not find an effect of boldness, nor an interaction between boldness with trip type on the habitat use of Cory’s shearwaters in relation to any dynamic environmental variable (Table 2). We also found no evidence for interacting effects of boldness with sex, or boldness with year in any habitat use variable (Table 2).

### Effect of boldness on trophic ecology

We did not find an effect of boldness on the trophic ecology of adult Cory’s shearwaters (Table 2). We also found no evidence for interacting effects of boldness with sex, or boldness with year on *δ*^13^C and *δ*^15^N values in both red blood cells and plasma (Table 2).

### Effect of boldness on chick growth

**Fig. 3:**
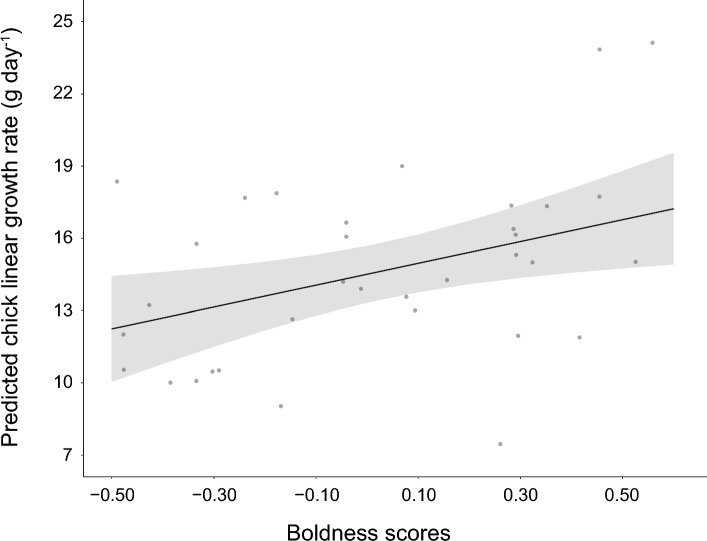
Mean predicted effect of parent’s boldness (NMDS 1 values) on the chick linear growth rate (g day^−1^). Lower NMDS 1 values represent ‘shyer’ responses and higher NMDS 1 values represent ‘bolder’ responses toward the object. Points represent predicted values. Predicted values (*points*), regression lines and 95% confidence intervals (*shaded areas*) were extracted from the best-supported models (models with the lowest Akaike’s information criterion corrected for sample sizes—AICc values; see Table 2)

Boldness was associated to an increase of 1.4 times in the chick linear growth rate (*β* ± SE: 4.53 ± 1.72, *F*_*1*,33_ = 6.92, *p* = 0.01), predicting that chicks raised by bolder parents grow at a faster rate during the linear growth phase (predicted chick linear growth rate: 17.2 g day^−1^, 95% CI = 14.9–19.6 g day^−1^) than those raised by shyer parents (predicted chick linear growth rate: 12.2 g day^−1^, 95% CI = 10.0–14.4 g day^−1^; Fig. 3). There was no evidence for interacting effects of boldness with sex, or boldness with year on chick linear growth rate (Table 2).

## Discussion

We found that chick-provisioning Cory’s shearwaters varied in their habitat use between short coastal trips (i.e., higher resource predictability) and long oceanic excursions (i.e., lower resource predictability), and that these variations were much smaller for bolder than for shyer individuals. Thus boldness may influence resource acquisition strategies, though the direction of these relationships may shift with different levels of resource predictability. Moreover, our findings are consistent with our predictions and recent research on breeding seabirds, demonstrating that shyer individuals typically exhibit greater foraging flexibility, whereas bolder birds are more behaviourally specialized (Harris et al. 2020a). Despite variations in foraging behaviour and habitat use between personality types, we found no evidence that bolder and shyer individuals had different dietary specializations. Yet, chicks raised by bolder parents grew at a faster rate than those raised by shyer parents. Our results suggest that the relationship between boldness and chick growth may be driven by diverse resource acquisition strategies, even when individuals reveal similar isotopic ecology. Together, these results suggest a potential mechanism through which boldness may influence breeding performance.

As predicted, bolder Cory’s shearwaters exhibited comparably lower foraging effort, suggesting different resource acquisition strategies along personality traits. Similar patterns were previously found by Patrick et al. (2017) for wandering albatrosses (*Diomedea exulans*), with the authors arguing that bolder individuals tend to favour a more risk-prone behaviour (Sih et al. 2004; Wolf et al. 2007; Dammhahn and Almeling 2012), likely foraging more efficiently, with shorter time spent foraging per trip (i.e., with less effort) (Patrick and Weimerskirch 2014). Indeed, bolder Cory’s shearwaters may benefit from high resource predictability to be comparably more efficient in searching for food resources (Cockrem 2022).

Boldness has been associated with different resource acquisition strategies in a number of species (Bergvall et al. 2011; Kurvers et al. 2012; Jolles et al. 2013; Keiser and Pruitt 2014), including seabirds (Patrick et al. 2017; Traisnel and Pichegru 2019). Our results support the hypothesis that boldness influences resource acquisition strategies, because bolder individuals exhibited larger foraging ranges and were more likely to forage in areas with higher and more variable bathymetry, thereby exhibiting an ability to explore the environment more widely (Wolf et al. 2007; Dammhahn and Almeling 2012). However, these relationships occurred during short coastal trips, but not during long oceanic forays. Bold, fast-explorer personalities and more risk-prone behaviours should be more successful in predictable environments, as these animals might dominate resources which are uniformly distributed (Dingemanse and Réale 2005; Cockrem 2007). Indeed, bolder Cory’s shearwaters may enlarge their foraging ranges during short trips to take advantage of predictable prey patches at the colony surroundings, and thus restrict shyer individual’s ability to acquire their preferred resources (Spiegel et al. 2017; Schirmer et al. 2019). This behaviour would enable bolder individuals to gain accurate information on the location and quality of prey patches, hence maximising their foraging efficiency, without much foraging effort. In a previous study on breeding black-browed albatross (*Thalassarche melanophrys*), Patrick and Weimerskirch (2014) found that bolder birds foraged in productive areas along the shelf edge near the breeding colony, where foraging efficiency and competition are expected to be higher, while shyer individuals avoided these regions, probably as a consequence of exploitative competition. In contrast to the previous study, Cory’s shearwaters exhibited different foraging ranges between personality types, while showing high spatial overlap in foraging areas, suggesting an absence of intra-specific competition, as supported by a previous study (Pereira et al. 2022). In fact, for this rather small population (800–975 breeding pairs; Oliveira et al. 2020), interference competition seems to only emerge under scenarios of low food availability (Haug et al. 2015; Paiva et al. 2017; Krüger et al. 2019). We instead suggest that the relationship between boldness and foraging ranges could be driven by differences in foraging flexibility, as a possible mechanism to adapt to resource availability between bolder and shyer individuals.

As expected, we showed that bolder birds tend to exhibit similar foraging ranges during both short and long trips, while shyer individuals greatly increased their foraging ranges from short to long trips. These results are in line with the existing literature demonstrating that bolder individuals generally exhibit inflexible and routine-like search patterns, while shyer individuals being more prone to display a greater behavioural flexibility and more variable habitat use (Benus et al. 1990; Wolf et al. 2008; Coppens et al. 2010). A study on breeding black-legged kittiwakes (*Rissa tridactyla*) by Harris et al. (2020a) reported that bolder birds exhibited higher foraging site fidelity when compared to shyer birds. The authors argued that the relationship between individual’s boldness and foraging flexibility could be driven by differences in habitat selection, i.e., if bolder and shyer individuals may chose habitat features varying in predictability, differences in spatial aspects of foraging may emerge. In agreement with this hypothesis, Sih (2013) showed that bolder individuals (i.e., with more specialized foraging behaviours) are likely to be more vulnerable to changes in their environments, as they are less able to exploit different habitats when the environmental conditions are unfavourable (Herborn et al. 2014).

In heterogenous environments, where resources vary in predictability (Weimerskirch 2007; Riotte-Lambert and Matthiopoulos 2020), bolder individuals may forage preferentially in areas which are more predictable over time (e.g., bathymetric features), while shyer individuals should be more prone to track ephemeral cues that change over short timescales (e.g., fronts and eddies). This appears to be the case in our study, as during long trips, when resources are less predictable than during short forays, bolder Cory’s shearwaters foraged more often in regions of lower bathymetry (likely shelf edges and seamounts), whereas shyer Cory’s shearwaters foraged more variably in regions of higher bathymetry, indicating greater flexibility when foraging in oceanic waters. By targeting spatio-temporally predictable prey patches during long oceanic trips, bolder birds are more likely to get resources more quickly and efficiently than shyer individuals. This strategy would allow bolder individuals to increase their overall fitness after successive chick-provisioning trips (Granadeiro et al. 1998), which in turn would positively influence their breeding success. Nevertheless, these results should be interpreted with caution, as the number of long trips represent about 10% of the total amount of trips in the two breeding seasons.

In contrast with our predictions, we found no significant association between individual’s boldness and their diet. Our results suggest that individual’s boldness can lead to variations in foraging behaviour and distribution, even when individuals consume resources with similar isotopic ecology. Cory’s shearwaters at Berlenga Island are known to feed on epipelagic fish (Paiva et al. 2010b; Alonso et al. 2012), which are likely to be associated with coastal upwelling in nearshore areas in the colony surroundings (Pereira et al. 2022), and therefore could be equally consumed by both bolder and shyer individuals. Moreover, birds from our study population spent little time foraging in the same areas as coastal fisheries, and spatial overlap with fishing vessels was not influenced by individual’s boldness (Pereira et al. 2021). Together, these results support the hypothesis that differences in foraging behaviour and spatial aspects of foraging between bolder and shyer Cory’s shearwaters from our study population was not likely driven by interference competition. Instead, we suggest that variations in foraging behaviour and habitat use may arise from differences in risk-taking behaviour, foraging flexibility tendencies and response to environmental cues.

We found that chicks raised by bolder parents grew at a faster rate than those raised by shyer parents. These results must be interpreted with caution, as only one parent from each nest was considered in this study. In addition, all the individuals used in this study were caught as breeding adults of unknown age. Future research should study the influence of boldness in foraging behaviour of Cory’ s shearwater in relation to age, as foraging ability tends to increase with breeding experience (Votier et al. 2017). Nevertheless, our results are in line with previous research demonstrating that boldness is correlated with variation in reproductive output, with bolder individuals often exhibiting greater reproductive success than shyer relatives (Smith and Blumstein 2008), including in breeding seabirds (Patrick and Weimerskirch 2014; Collins et al. 2019; Harris et al. 2020b). Yet, our results contrast with those reported for breeding African penguins (*Spheniscus demersus*), showing that chicks raised by bolder parents grew significantly slower than those raised by shyer parents, especially in years of low resource availability (Traisnel and Pichegru 2018). One possible explanation for the contrasting results from our study and those in Traisnel and Pichegru (2018) could be related to the ability that different personality types have to cope with variations in resource predictability. Bolder individuals are likely to be more successful in environments where resources are spatio-temporal predictable (Dingemanse and Réale 2005; Cockrem 2007) and during years of good environmental conditions (Patrick and Weimerskirch 2014). Hence, we suggest that the positive relation between parent’s boldness and chick linear growth may be driven by differences in resource acquisition strategies between bolder and shyer Cory’s shearwaters, with bolder individuals possibly being more efficient in resource acquisition than shyer individuals. By decreasing foraging effort, and likely being more successful in resource acquisition, bolder individuals may allocate more energy to their reproductive effort (Smith and Blumstein 2008; Hollander et al. 2008; Careau et al. 2008).

To conclude, we show that bolder and shyer Cory’s shearwaters exhibited different foraging behaviour and habitat use, with bolder individuals likely being more efficient in resource acquisition than shyer counterparts. In addition, bolder birds were more consistent in their habitat than shyer individuals, indicating that bolder birds were more behavioural specialized whereas shyer birds were more flexible in their behaviour. We hypothesize that differences in resource acquisition strategies may be a mechanism through which boldness may influence breeding performance.

### Supplementary Information

Below is the link to the electronic supplementary material.

Supplementary file1 (DOCX 638 KB)

## Data Availability

GPS tracking data are available at the BirdLife International Seabird Tracking Database (http://www.seabirdtracking.org) under ID 1059.
