# Supplementary material for: Boldness predicts foraging behaviour, habitat use and chick growth in a central place marine predator
Source: Oecologia. 2024 May 13;205(1):135–47. doi: 10.1007/s00442-024-05557-4 (PMC11144154; doi:10.1007/s00442-024-05557-4)
Supplement: Supplementary file 1 — Supplementary file1 (DOCX 638 KB) [file 442_2024_5557_MOESM1_ESM.docx]

***The following supplement accompanies the article***

BOLDNESS PREDICTS FORAGING BEHAVIOUR, habitat use AND CHICK GROWTH IN A CENTRAL PLACE MARINE PREDATOR

Jorge M. Pereira^1,*^, Jaime A. Ramos^1^, Filipe R. Ceia^1^, Lucas Krüger^2,3^, Ana M. Marques^1^ and Vitor H. Paiva^1^

*^1^ University of Coimbra, MARE – Marine and Environmental Sciences Centre / ARNET - Aquatic Research Network, Department of Life Sciences, Calçada Martim de Freitas, 3000-456 Coimbra, Portugal*

*^2^ Instituto Antártico Chileno, Plaza Muñoz Gamero 1055, Punta Arenas 620 000, Chile*

*^3^ Instituto Milenio Biodiversidad de Ecosistemas Antárticos y Subantárticos (BASE), Las Palmeras 3425, Ñuñoa, Santiago, Chile*

* Corresponding author: [jorge.pereira@uc.pt](mailto:jorge.pereira@uc.pt)

***Supplementary Material 1: Detailed description of GPS tag deployments***

GPS-loggers were programmed to record positions at 10-min intervals in 2017 and 5-min intervals in 2018. Different time intervals were set in the two years to ensure sufficient battery life to record multiple trips per bird in a previous study comparing the foraging behaviour between individuals from different sub-colonies during short and long trips (Pereira et al. 2022). Tracking duration was therefore higher in 2017 than in 2018 (Table 1). However, we believe that these differences in tracking duration did not influenced our results, given that: (1) all analyses on tracking data were conducted separately for each year (see “Material and methods” for full details on the analyses); and (2) foraging trip characteristics (i.e. trip duration, maximum distance from the colony and proportion of long trips) were similar between both years (see Table 1 and results on “Foraging trip characteristics”). GPS loggers weighed approximately 17 g, which was 2.6% of body mass of the lightest bird tagged. We detected no significant decrease in body mass of tagged individuals between capture (868.1 ± 99.4 g) and recapture (865.3 ± 94.8 g; F_1,33_= 0.01, *p*= 0.91).

***Supplementary Material 2: Laboratory procedures for stable isotope analysis***

Approximately 0.3 mg of each sample was weighted in a tin capsule using an ultra-microbalance. Values of *δ*^15^N and *δ*^13^C were determined using a Continuous Flow Isotope Ratio Mass Spectrometer (Delta V™ Advantage - Thermo Scientific®) with an organic elemental analyser (Flash™ EA 1112 - Thermo Scientific®). Results are expressed in delta (*δ*) notation as parts per thousand (‰) and calculated using the equation *δ*X = (Rsample – Rstandard – 1) x 1000, where X is N or C and R the ratio between ^15^N/^14^N and ^13^C/^12^C. Atmospheric N_2_ and Vienna-Pee Dee Belemnite Limestone were use as standard for N and C respectively. Acetanilide (Thermo Scientific®) was used as reference material to measure machine internal errors (< 0.3 ‰ for *δ*^15^N and < 0.1 ‰ for *δ*^13^C).

**Table S1.** Variable loadings and stress-value from a non-metric multidimensional scaling (NMDS) analysis of 6 behaviours measured from 35 Cory’s shearwaters (*Calonectris borealis*), as a response and/or aggression towards a novel object at the nest. Stress-value represent the extent to which a two-dimensional plot is accurate in summarising the separation of observations, with values lower than 0.2 indicating a good NMDS analysis (Clarke 1993).

| **Behaviour** | **NMDS 1 values** | **NMDS 2 values** |
| --- | --- | --- |
|  |  |  |
| Head | -0.30 | 0.10 |
| Spasm | -0.15 | -0.49 |
| Snap | 0.12 | -0.03 |
| Move | 0.17 | -0.35 |
| Peck | 0.29 | -0.10 |
| Stand | 0.66 | -0.13 |
| Stress value | 0.03 | |

| **Type** | **Environmental variable** | **Data source** | **Data resolution  (temporal/ spatial)** | **Rationale for inclusion** |
| --- | --- | --- | --- | --- |
|  |  |  |  |  |
| Static | Bathymetry  (m) | Raster file was downloaded from:  <https://www.ngdc.noaa.gov/mgg/global/global.html> | 0.01° | Proxy of prey predictability. Shallow bathymetries (e.g. seamounts, shelf breaks and continental shelves) often correlate with foraging habitats that are spatio-temporal predictable, which in turn drive large aggregations of seabirds (Wakefield et al. 2021), including Cory’s shearwaters (Paiva et al. 2010a; Alonso et al. 2018). |
| Dynamic | Chlorophyll-*a* concentration  (mg m^-3^) | Raster files were downloaded from:  <http://marine.copernicus.eu> | 8 days/ 0.08° | Proxy of prey concentration. Foraging shearwaters often select areas of high primary productivity (Navarro and González-Solís 2009; Paiva et al. 2010b; Pereira et al. 2022). |
| Dynamic | Ocean mixed layer depth  (m) | Raster files were downloaded from: <http://marine.copernicus.eu> | 8 days/ 0.08° | Thickness of the mixed layer depth is greatly influenced by upwelling of nutrient-rich and cold waters into the mixed layer, affecting localised primary productivity and seabird prey availability (Serratosa et al. 2020; Cerveira et al. 2020). |
| Dynamic | Eddy kinetic energy  (cm-^2^ s^-2^) | Eddy kinetic energy was derived from the zonal (𝑈) and meridional (𝑉) sea water velocities, and calculated following the equation:  1/2 (𝑈^2^ + 𝑉^2^), where 𝑈 and 𝑉  Raster files were downloaded from <http://marine.copernicus.eu> | 8 days/ 0.08° | Proxy of oceanographic processes favouring water mixing and nutrient influx, ultimately boosting prey aggregation in frontal zones or eddy edges (Wakefield et al. 2009; Pereira et al. 2020; De Pascalis et al. 2021) |

**Table S2.** Description of the explanatory environmental variables used in the best-supported models (models with the lowest Akaike’s information criterion corrected for sample sizes - AICc values) and the rationale for their inclusion.

**Table S3.** Variance inflation factors (VIF) used to inspect collinearity among environmental variables. Variables with VIF values ≥ 2.5 indicate considerable collinearity (Johnston et al. 2018), and therefore were excluded from the model selection.

|  | **VIF values** |
| --- | --- |
|  |  |
| Bathymetry (m) | 2.0 |
| Chlorophyll-*a* concentration (mg m^-3^) | 1.3 |
| Sea surface temperature (°C) | 7.7 |
| Sea surface height (m) | 8.1 |
| Ocean mixed layer depth (m) | 1.4 |
| Eddy kinetic energy (cm^-2^ s^-2^) | 2.3 |

**Figure S1.** Frequency distributions of trip duration (days) and maximum distance from the colony (km) for 314 trips made by 35 Cory’s shearwaters (*Calonectris borealis*) at Berlenga Island over two breeding seasons (2017-2018), during chick-rearing. Short trips were defined as ≤ 2 days and ≤ 100 km, and long trips as > 2 days and > 100 km.


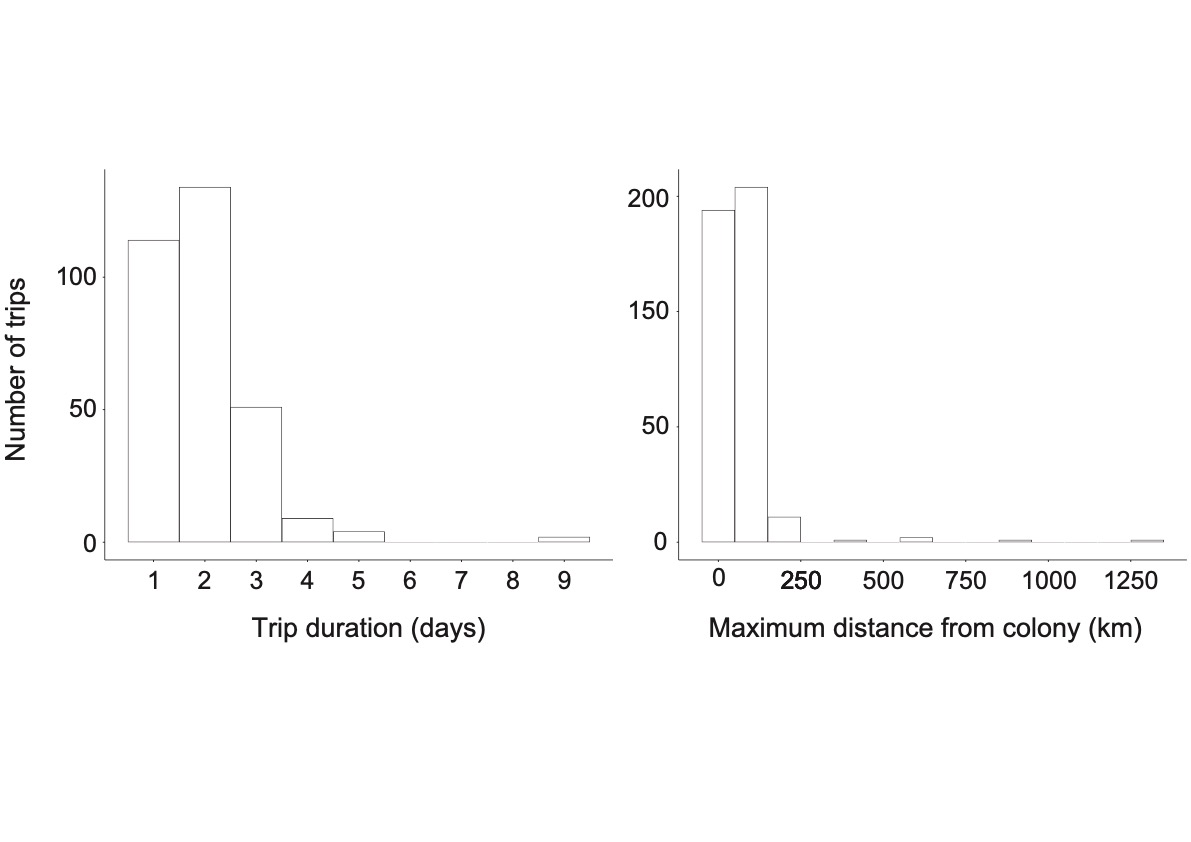


**Figure S2.** Overall foraging trips made by Cory’s shearwaters at Berlenga Island during the chick-rearing period (2017-2018), overlaid on the bathymetry of the region. Location of the breeding colony is marked with a yellow star.


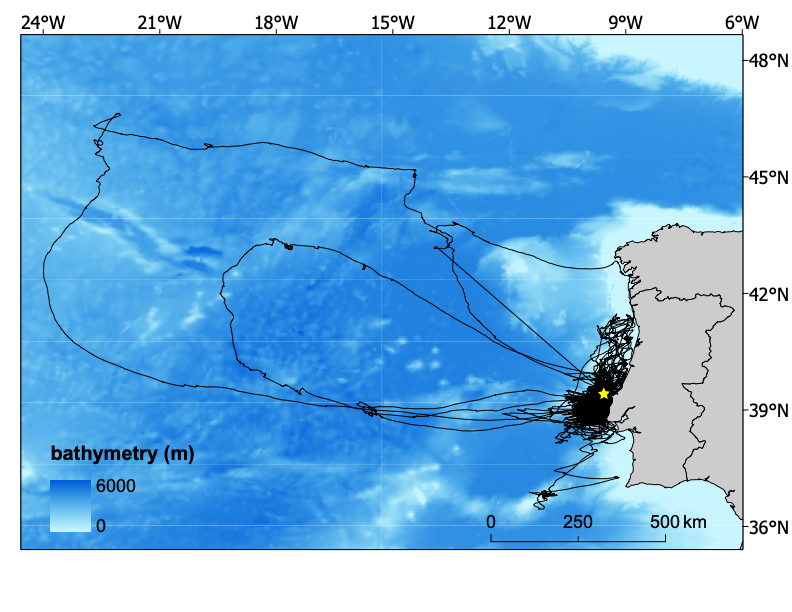


**Figure S3.** Mean predicted effect of individual’s boldness (NMDS 1 values) on the proportion of time spent foraging (%) interacting with trip type (short *vs*. long trips). Red lines represent short trips and blue lines represent long trips. Lower NMDS 1 values represent ‘shyer’ responses and higher NMDS 1 values represent ‘bolder’ responses toward the object. Regression lines and 95% confidence intervals (shaded areas) were extracted from the best-supported models (models with the lowest Akaike’s information criterion corrected for sample sizes - AICc values).

**
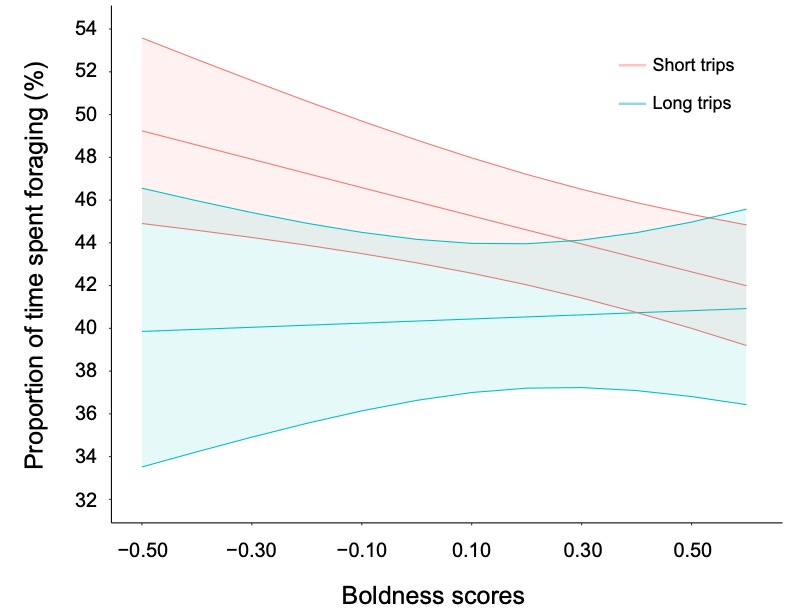
**

**References**

Alonso H, Granadeiro JP, Dias MP, et al (2018) Fine-scale tracking and diet information of a marine predator reveals the origin and contrasting spatial distribution of prey. Progress in Oceanography 162:1–12. https://doi.org/10.1016/j.pocean.2018.02.014

Cerveira LR, Ramos JA, Rodrigues I, et al (2020) Inter-annual changes in oceanic conditions drives spatial and trophic consistency of a tropical marine predator. Marine Environmental Research 162:105165. https://doi.org/10.1016/j.marenvres.2020.105165

Clarke KR (1993) Non‐parametric multivariate analyses of changes in community structure. Australian Journal of Ecology 18:117–143. https://doi.org/10.1111/j.1442-9993.1993.tb00438.x

De Pascalis F, Pala D, Pisu D, et al (2021) Searching on the edge: dynamic oceanographic features increase foraging opportunities in a small pelagic seabird. Marine Ecology Progress Series 668:121–132. https://doi.org/10.3354/meps13726

Johnston R, Jones K, Manley D (2018) Confounding and collinearity in regression analysis: a cautionary tale and an alternative procedure, illustrated by studies of British voting behaviour. Quality & Quantity 52:1957–1976. https://doi.org/10.1007/s11135-017-0584-6

Navarro J, González-Solís J (2009) Environmental determinants of foraging strategies in Cory’s shearwaters Calonectris diomedea. Marine Ecology Progress Series 378:259–267. https://doi.org/10.3354/meps07880

Paiva VH, Geraldes P, Ramírez I, et al (2010a) How area restricted search of a pelagic seabird changes while performing a dual foraging strategy. Oikos 119:1423–1434. https://doi.org/10.1111/j.1600-0706.2010.18294.x

Paiva VH, Geraldes P, Ramírez I, et al (2010b) Oceanographic characteristics of areas used by Cory’s shearwaters during short and long foraging trips in the North Atlantic. Marine Biology 157:1385–1399. https://doi.org/10.1007/s00227-010-1417-5

Pereira JM, Paiva VH, Ceia FR, Ramos JA (2020) Facing extremes: Cory’s shearwaters adjust their foraging behaviour differently in response to contrasting phases of North Atlantic Oscillation. Regional Environmental Change 20:77. https://doi.org/10.1007/s10113-020-01662-1

Pereira JM, Ramos JA, Almeida N, et al (2022) Foraging costs drive within-colony spatial segregation in shearwaters from two contrasting environments in the North Atlantic Ocean. Oecologia 199:13–26. https://doi.org/10.1007/s00442-022-05109-8

Serratosa J, Hyrenbach KD, Miranda-Urbina D, et al (2020) Environmental Drivers of Seabird At-Sea Distribution in the Eastern South Pacific Ocean: Assemblage Composition Across a Longitudinal Productivity Gradient. Frontiers in Marine Science 6:. https://doi.org/10.3389/fmars.2019.00838

Wakefield E, Phillips R, Matthiopoulos J (2009) Quantifying habitat use and preferences of pelagic seabirds using individual movement data: a review. Marine Ecology Progress Series 391:165–182. https://doi.org/10.3354/meps08203

Wakefield ED, Miller DL, Bond SL, et al (2021) The summer distribution, habitat associations and abundance of seabirds in the sub-polar frontal zone of the Northwest Atlantic. Progress in Oceanography 198:102657. https://doi.org/10.1016/j.pocean.2021.102657
